# Geospatial Distribution of Ambulatory Surgery Center Utilization for Otorhinolaryngologic Surgeries Among Medicare Patients From 2015 to 2019

**DOI:** 10.1002/oto2.57

**Published:** 2023-06-08

**Authors:** Mark Nyaeme, Rahul S. Yerrabelli, Nicholas Peterman, Brad Kaptur, Eunhae Yeo, Kristine R. Carpenter

**Affiliations:** ^1^ Carle Illinois College of Medicine University of Illinois at Urbana‐Champaign Champaign Illinois USA; ^2^ Carle Foundation Hospital Urbana Illinois USA

**Keywords:** ambulatory surgery, cost, CPT code, demographics, ENT, geography, geospatial analysis, HCPCS code, Moran's I, otolaryngology, otorhinolaryngology, outpatient surgery, spatial autocorrelation, USA

## Abstract

**Objective:**

To investigate the geographic clustering of ambulatory surgical center (ASC) utilization in otolaryngology to determine hot spot areas of high utilization and cold spot areas of low utilization and socioeconomic factors that correlate with these hot spots and cold spots.

**Study Design:**

To develop a national epidemiologic study of ASC utilization in otolaryngology in the United States.

**Setting:**

United States of America.

**Methods:**

Multiple county‐level national databases were reviewed including Center for Medicare Services (CMS) physician billing data, CMS Medicare demographic data, and US Census socioeconomic data. The analysis was conducted using the average of all Medicare billing information from 2015 to 2019. Whether a procedure was performed in an ASC was extracted from CMS data using the CMS definition of an ASC. The percentage ASC billing was calculated as the fraction of CMS payments that were performed in ASCs for the top ENT procedures. A Python‐based script for database building and GeoDa, Moran's I clustering coefficient, and a 1‐way analysis of variance was utilized to chart and analyze demographic, geographic, and socioeconomic trends.

**Results:**

Hot spots of utilization, with an average ASC billing of 80.13%, were seen in Southern California, Florida, Mid‐Atlantic, and clusters throughout the Deep South. Cold spot clusters, with an average ASC billing of 2.21%, were located in large swaths of New England, Ohio, and the Deep South with clusters bisecting the Midwest. Cold spots had a higher percentage of poverty and percent eligible for Medicaid.

**Conclusion:**

ASC utilization is best used to improve cost‐effectiveness and accessibility of care but what is seen is that ASC use is currently highest in cities in coastal areas which already have high levels of care access and are making the most proportional money compared to their rural counterparts.

Since the 1980s, advancements in anesthesia and surgical care have made possible the shift of many types of surgical interventions from the inpatient setting to the outpatient (ambulatory) setting. In addition, these outpatient surgeries and procedures are less frequently occurring in hospital outpatient departments (HOPDs), facilities owned and generally attached to a hospital, and more often occurring in ambulatory surgery centers (ASCs), typically standalone facilities that are dedicated solely to high‐throughput of same‐day surgical care. Proponents have embraced the greater efficiency of these centers and their ability to significantly reduce costs to the health care system, while others have questioned the safety of moving an expanding number of procedures and procedure types to these centers.

In the United States, estimates for the fraction of surgeries done in the ambulatory setting vary greatly from 52% to 80% depending on the inclusion criteria.^1^, ^2^, ^3^ Within otolaryngology, there are several surgeries that are usually or almost exclusively done in the ambulatory setting including tympanoplasty (97.4%), myringotomy (97.3%), mastoidectomy (87.2%), tonsillectomy and/or adenoidectomy (95.5%), plastic surgery procedures on nose (91.9%), and other nose, mouth, pharynx, and ear procedures (65.6%‐69.7%).^2^ The use of ambulatory surgical centers allows for great convenience for the patient. The mean length of stay for the ambulatory setting was 0.2 days while the inpatient mean was 6 days. On top of that, on average a procedure done in the ASC setting takes 31.8 minutes less than their inpatient counterparts.^3^ These factors combined make the utilization of ASC an efficient and economical setting for procedures, especially as future demand increases.

Ophthalmology (36%), orthopedics (36%), and gastroenterology (32%) are the specialties that have embraced the transition to ASC most strongly and have the highest percentage of procedures done at these centers. Otolaryngology has also progressively transitioned to ASCs due to cost and accessibility benefits for the patients.

The goal of this study is to investigate the geographic clustering of ASC utilization in otolaryngology to determine hot spots of high utilization and socioeconomic factors that correlate with these hot spots and cold spots.

## Methods

### Data Sources, Collection, and Extraction

National, publicly available databases from the US government were combined for 2015 to 2019 to encompass the Medicare billing patterns of ear, nose, and throat (ENT) practitioners as well as the corresponding Medicare populations for each applicable county in the continental United States. Databases included: Center for Medicare Services (CMS) physician billing data,^4^ CMS Medicare demographic data,^5^ and US Census socioeconomic data.^6^ Due to using only publicly available data, institutional review board or ethics committee approval was not applicable for this study. First, a list of the top 100 ENT procedure codes was extracted from the “Coding Corner,” a resource from the American Academy of Otolaryngology–Head and Neck Surgery (AAO‐HNS) to teach CMS coding to its members. For the list, AAO‐HNS's “top 100” criteria was the total CMS billing amount for those instances of the procedure codes done in an ASC. We started with a list from AAO‐HNS to ensure the procedure codes we studied were limited to those in the ENT specialty. Second, the aforementioned CMS physician billing data set^4^ was then used to obtain information on these 100 procedure codes including the geographic locations they were performed, the total number of procedure services, and the fraction of the services performed at an ASC versus a HOPD. Third, the list of 100 procedure codes was further filtered to the 53 procedure codes that were performed at least once in an HOPD and at least once in an ASC. In this article, we use the term procedure codes to refer to CMS's Health Care Common Procedure Coding System (HCPCS codes) which are synonymous with Current Procedural Terminology codes in this context.

Multiple linear regressions were conducted across each of the studied variables for each county across the 5 years of billing between 2015 and 2019 to identify how these variables changed over time at a county‐level granularity. Counties without billing of the chosen procedures in the time frame were removed from the analysis. The data set on Medicare demographic data^5^ allowed for the county‐level approximation of the Medicare members within each billing area. Census data was used to identify population‐level socioeconomic variables to further supplement the data set. Each database was grouped at a county level and then combined utilizing Python with GeoDa, a geospatial analysis software, used for map‐based hot spot analysis and visualization.^7^, ^8^

We use the term county to indicate either legally‐defined counties or “county‐equivalents,” which is a term used by the US Census Bureau that also includes certain edge cases: for example, Baltimore, MD and Washington, DC, which are not legally inside any county, and parishes, which Louisiana's legal equivalent to counties.

### Statistical Analysis

Cluster analysis in GeoDa was conducted using Moran's I statistic.^9^ Moran's I allow for a measure of significant spatial variation between counties across a variable of interest. The statistic groups each county into 1 of 4 statistically significant classifications for a variable of interest: high‐high, low‐low, low‐high, and high‐low. The first of the 2‐part classification describes if a county's value of interest is statistically significantly higher or lower than the national average. The second part of the classification describes if a county's average neighbor's value of interest is significantly higher or lower than the national average. If both a county and its average neighbor are significant in either direction, then the county as a whole is significant according to Moran's I statistic. High‐high and low‐low grouping represent hot spots and cold spots respectively, while low‐high and high‐low groupings demarcate geospatial areas of incongruence. The neighbor designation is determined by the minimum distance between the centroids of counties to allow for each county to have at least 1 neighbor, which is approximately 100 miles.

The 4 significant Moran's I groupings of ambulatory surgical center utilization were then further analyzed using a 1‐way analysis of variance across all county‐level variables to identify disparities. A 2‐tailed *t* test was then used to compare only high‐high (hot spot) and low‐low (cold spot) groupings.

## Results

### National Level Statistics

After the removal of counties without billing data between 2015 and 2019, 941 counties in the continental United States remained. While this is only 29.9% of the 3142 counties in the United States, the included counties comprise a total population of 268 million, which is approximately 84% of the US population. Every state in the continental US had multiple counties that were included. The 8 states with the greatest fraction of counties included were all in the Northeast or Middle Atlantic region (DE: 3/3, NJ: 20/21, RI: 4/5, CT: 6/8, MA: 10/14, PA: 47/67, NH: 7/10, NY: 40/62 counties). Nebraska (8/93) and North Dakota (5/53) were the only states with less than 10% of counties included. However, these states still had the majority of their populations included (NE: 1,124,839 or ∼57% of the population, ND: 446,012 or ∼58% of the population). This is logical as the counties with insufficient CMS data to be included in our analysis were generally sparsely populated.

Of the top 100 billed procedure types, 53 were shared between ASC and HOPD facilities and thus were included in the analysis. The list of procedure types was diverse including “Diagnostic examination of voice box using flexible endoscope” to “Implantation of cochlear device” (Table 1; Supplemental File 1, available online). The 53 procedure types could be generally categorized into 1 of 5 ENT subspecialties: 11 in facial plastics, 9 in head and neck surgery, 6 in otology, 17 in rhinology, and 10 in laryngology (10) (Table 2).

**Table 1:** The Top 15 ENT Procedures by Medicare Payment With Linear Regression Analysis of Key Metrics From 2015 to 2019

	HCPCS information		Annual Medicare payment (thousands of USD/y)			Annual number of services			% ASC billing				
#	Code	Description	5‐y mean	Yearly change	*R* of fit	5‐y mean	Yearly change	*R* of fit	5‐y mean, %	Yearly change	*R* of fit	% of procedures performed in ASC	Ratio of ASC % billing to % procedure
1	14060	Tissue transfer repair of wound (≤10 cm²) of eyelids, nose, ears, and/or lips	7078	−154.9	−0.64	13,393	−335	−0.93	86.0	0.21	0.29	83.3	1.03
2	69930	Implantation of cochlear device	6596	1422.9	0.98	2180	287	0.98	71.6	2.79	0.96	9.4	7.62
3	31267	Removal of nasal sinus tissue using an endoscope	4374	680.9	0.92	8895	596	0.98	82.6	3.33	0.81	50.5	1.63
4	30520	Reshaping of nasal cartilage	4122	316.5	0.88	10,048	366	0.93	51.5	−1.04	−0.61	42.1	1.22
5	30140	Removal of nasal air passage (submucosal resection of inferior turbinate)	4035	−153.0	−0.46	12,283	1105	0.99	61.7	8.90	0.87	53.0	1.16
6	15260	Relocation of patient skin to nose, ears, eyelids, and/or lips (20 cm² or less)	3899	−99.9	−0.51	7004	−250	−0.89	87.9	0.50	0.53	88.9	0.99
7	14301	Tissue transfer repair of wound (30.1‐60.0 cm²)	3848	294.8	0.95	4640	204	0.98	79.4	0.14	0.10	68.1	1.17
8	14040	Tissue transfer repair of wound (10 cm² or less) of the forehead, cheeks, chin, mouth, neck, underarms, genitals, hands, and/or feet	3772	−162.2	−0.92	7340	−421	−0.91	87.4	0.23	0.40	83.2	1.05
9	31575	Diagnostic examination of voice box using flexible endoscope	3320	−222.2	−0.76	61,525	−1343	−0.44	0.1	0.04	0.71	0.1	0.97
10	31276	Exploration of nasal sinus using an endoscope	3095	−560.1	−0.69	5239	−707	−0.68	55.6	5.23	0.97	39.8	1.40
11	14041	Tissue transfer repair of wound (10.1‐30.0 cm²) of the forehead, cheeks, chin, mouth, neck, underarms, genitals, hands, and/or feet	2359	21.6	0.24	4188	−2	−0.01	85.6	0.75	0.49	84.1	1.02
12	60500	Removal or exploration of parathyroid glands	1669	191.6	0.86	2109	177	0.80	17.8	2.87	0.92	8.9	2.00
13	31231	Diagnostic examination of nasal passages using an endoscope	1624	86.2	0.81	35,839	2394	0.87	0.1	0.00	0.02	0.1	0.74
14	31579	Examination to assess movement of vocal cord flaps using an endoscope	1200	31.1	0.26	12,102	916	0.71	0.1	0.00	−0.16	0.1	0.53
15	69436	Incision of eardrum with insertion of eardrum tube under general anesthesia	1176	−105.1	−0.70	4086	−79	−0.69	78.5	−0.57	−0.34	55.6	1.41

Mean is the average across the 5 years. Change indicates the annual change as determined from the slope of the regression line. *R* is the Pearson correlation coefficient between the fitted linear regression and the data.

Abbreviations: ASC, ambulatory surgery center; HCPCS, Health Care Common Procedure Coding System; *R*, Pearson correlation coefficient; USD, $ or US dollar.

**Table 2:** The ENT Subspecialty Procedures by Medicare Payment With Linear Regression Analysis of Key Metrics From 2015 to 2019

	Annual Medicare payments in thousands of USD/y			The annual number of services		
Specialty with included HSPCS codes	5‐y mean	Yearly change, mean ± SE	*R* of fit	5‐y mean	Yearly change, mean ± SE	*R* of fit
Facial plastics (n = 11), codes: 14060, 14301, 14040, 14041, 30520, 15260, 11042, 15004, 20926, 15120, 15730	27,533	553 ± 238.0	0.802	61,439	472.9 ± 1304	0.205
Head and neck surgery (n = 9), codes: 11642, 11643, 60240, 60220, 60500, 31541, 42415, 38510, 42826	3374	83 ± 76.8	0.531	9435	−254.1 ± 119	−0.778
Otology (n = 6), codes: 69436, 69433, 69930, 21235, 69631, 69801	8691	139 ± 132.5	0.987	9960	83.3 ± 102	0.425
Rhinology (n = 17), codes: 31253, 31257, 30140, 30130, 31240, 31267, 31288, 31276, 31231, 31256, 31237, 30465, 31254, 30930, 31259, 30802, 31238	17,912	735 ± 514.2	0.637	77,483	4537.8 ± 596	0.975
Laryngology (n = 10), codes: 31536, 31535, 43191, 43200, 31575, 31579, 31525, 31526, 31571, 31622	6551	−36 ± 204.7	−0.102	88,304	482.4 ± 2232	0.124
All (n = 53)	64,063	2730 ± 33.6	1.000	246,620	5322.3 ± 2020	0.836

Mean is the average across the 5 years. The change indicates the annual change as determined by the slope of the regression line. *R* is the Pearson correlation coefficient between the fitted linear regression and the data.

Abbreviations: ASC, ambulatory surgery center; ENT, ear, nose, throat; HCPCS, Health Care Common Procedure Coding System; *R*, Pearson correlation coefficient; SE, standard error; USD, $ or US dollar.

**Figure 1:**
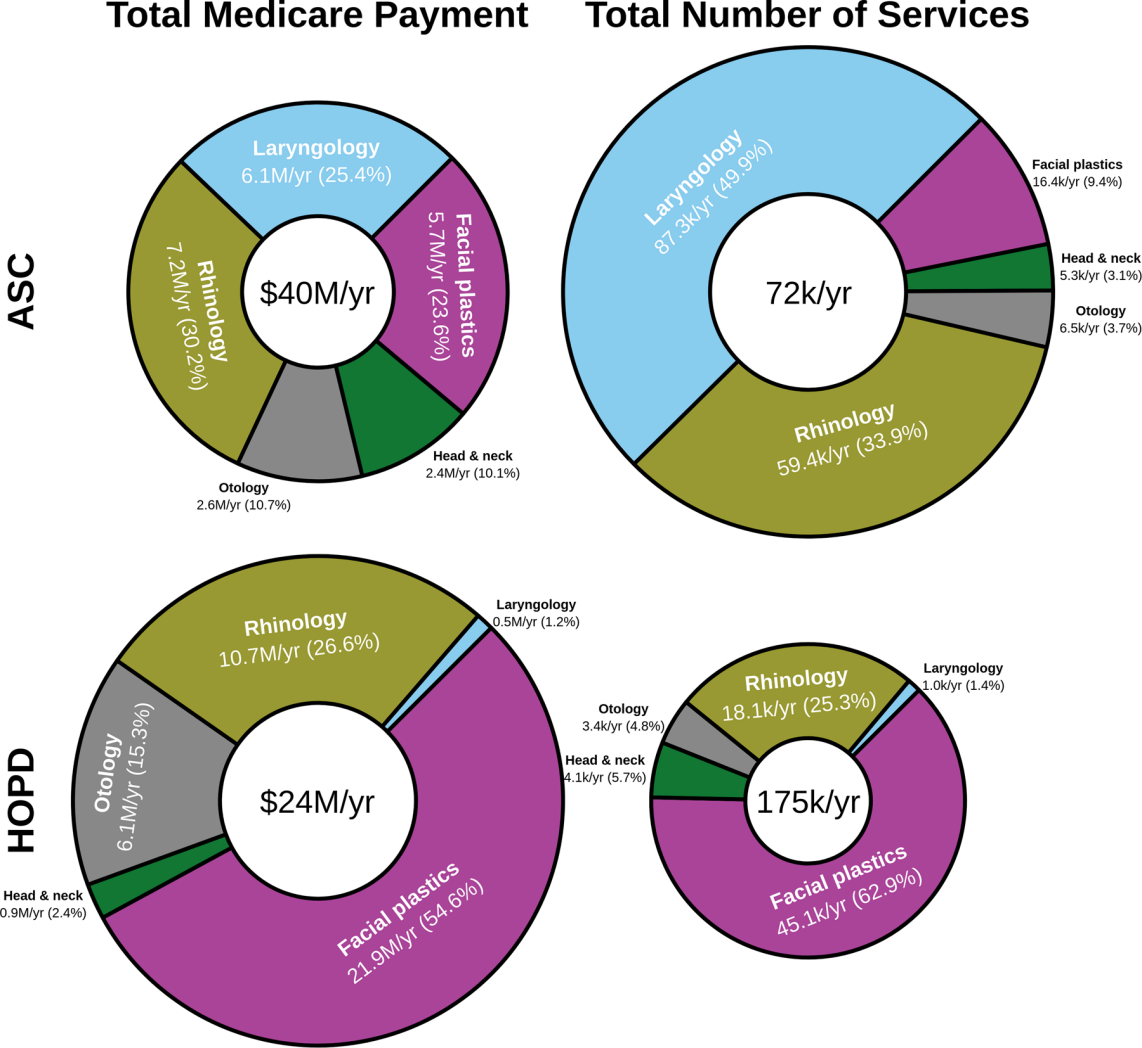
Pie chart describing the fraction of Medicare payments and services performed for each ear, nose, and throat subspecialty. The top row represents the values for ambulatory surgery centers (ASCs), while the bottom represents the values for hospital outpatient departments (HOPD).

**Figure 2:**
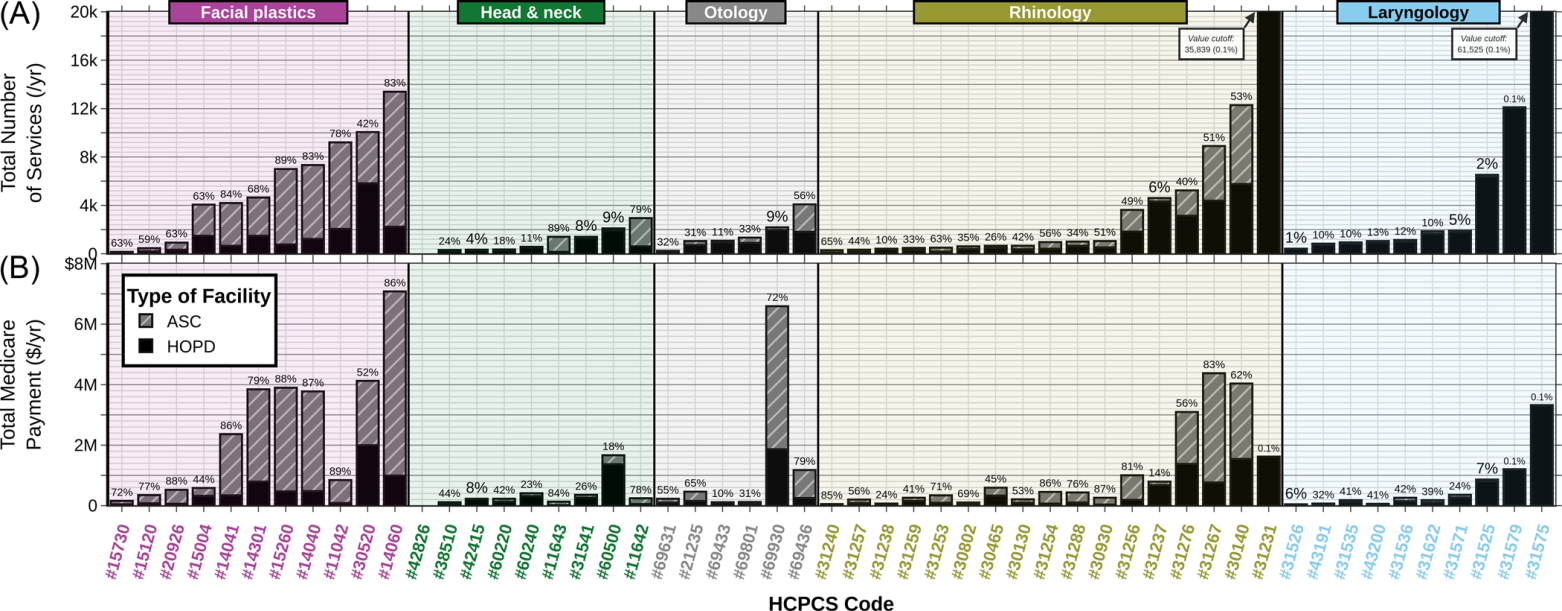
Bar chart describing the (A) services performed per year for each HCPCS code and (B) total Medicare payment. The bars are split divided into HOPD and ASC, and the proportion of ASC is written above each bar. Within each ear, nose, and throat subspecialty, the HCPCS codes are displayed in ascending order by number of services. ASC, ambulatory surgery center; HCPCS, Health Care Common Procedure Coding System; HOPD, hospital outpatient department.

**Figure 3:**
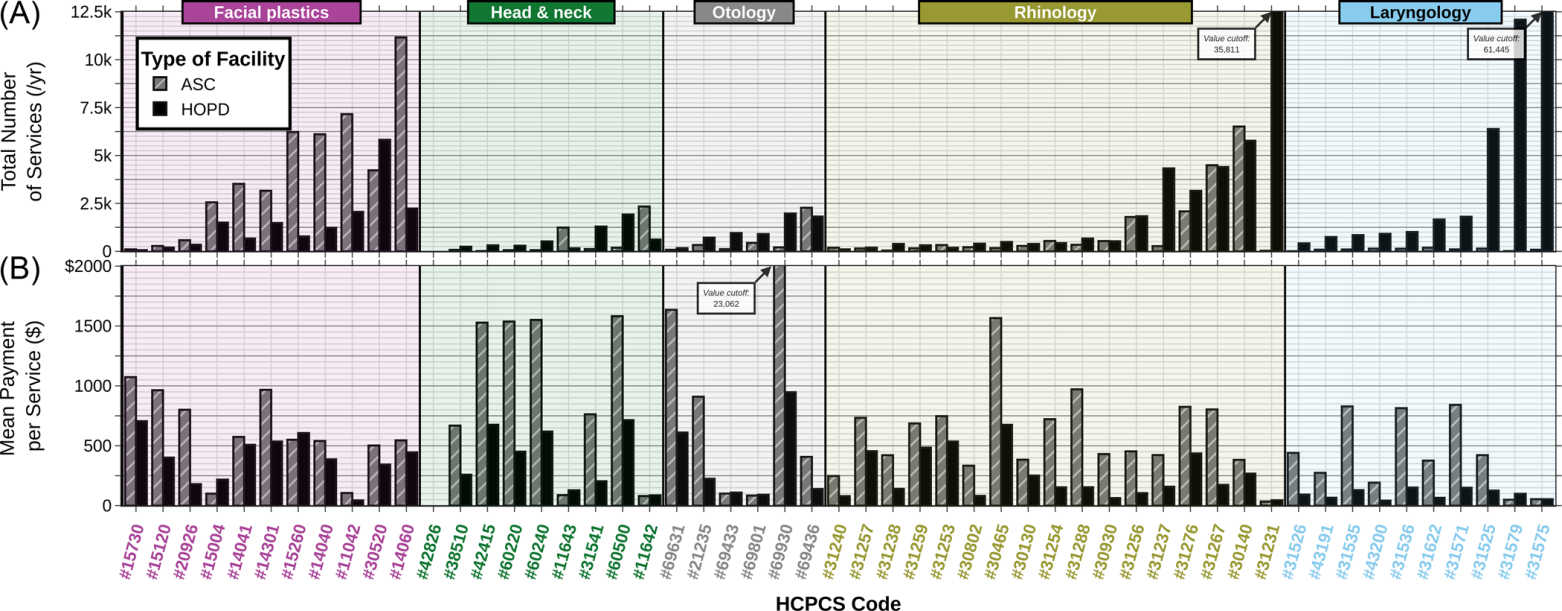
Bar chart comparing ASC and HOPD facilities by (A) the number of services performed and (B) the cost (Medicare payment) per service for each of the HCPCS codes. The payment per service was found by dividing by the total number of payments by the total number of services in a category. The figure shows that the payment per service was higher in ASCs for most HCPCS codes. ASC, ambulatory surgery center; HCPCS, Health Care Common Procedure Coding System; HOPD, hospital outpatient department.

A total of 1,233,099 procedures were found over the 5‐year period. These procedures had a corresponding total Medicare payment amount of $320,317,240, which makes an average annual cost of $64,063,448 (Figure 1). The costs increased an average of $2,730,352 (standard error [SE] $33,613) or 4.26% (SE 0.052%) per year. While ASCs only had 29.08% of the noted procedures, they comprised 62.54% of the total Medicare payment (Figures 1 and 2). The Medicare payment per service performed was higher in ASCs than HOPDs in all of the ENT subspecialties and in most of the HCPCS codes. This trend was also true regardless of how frequently or infrequently the procedure was performed in ASCs versus HOPDs (Figure 3). The percent billing toward ASC increased by an average of 2.32% each year with the total ASC increasing by 0.1% on average per year.

### Geographic Clustering

**Figure 4:**
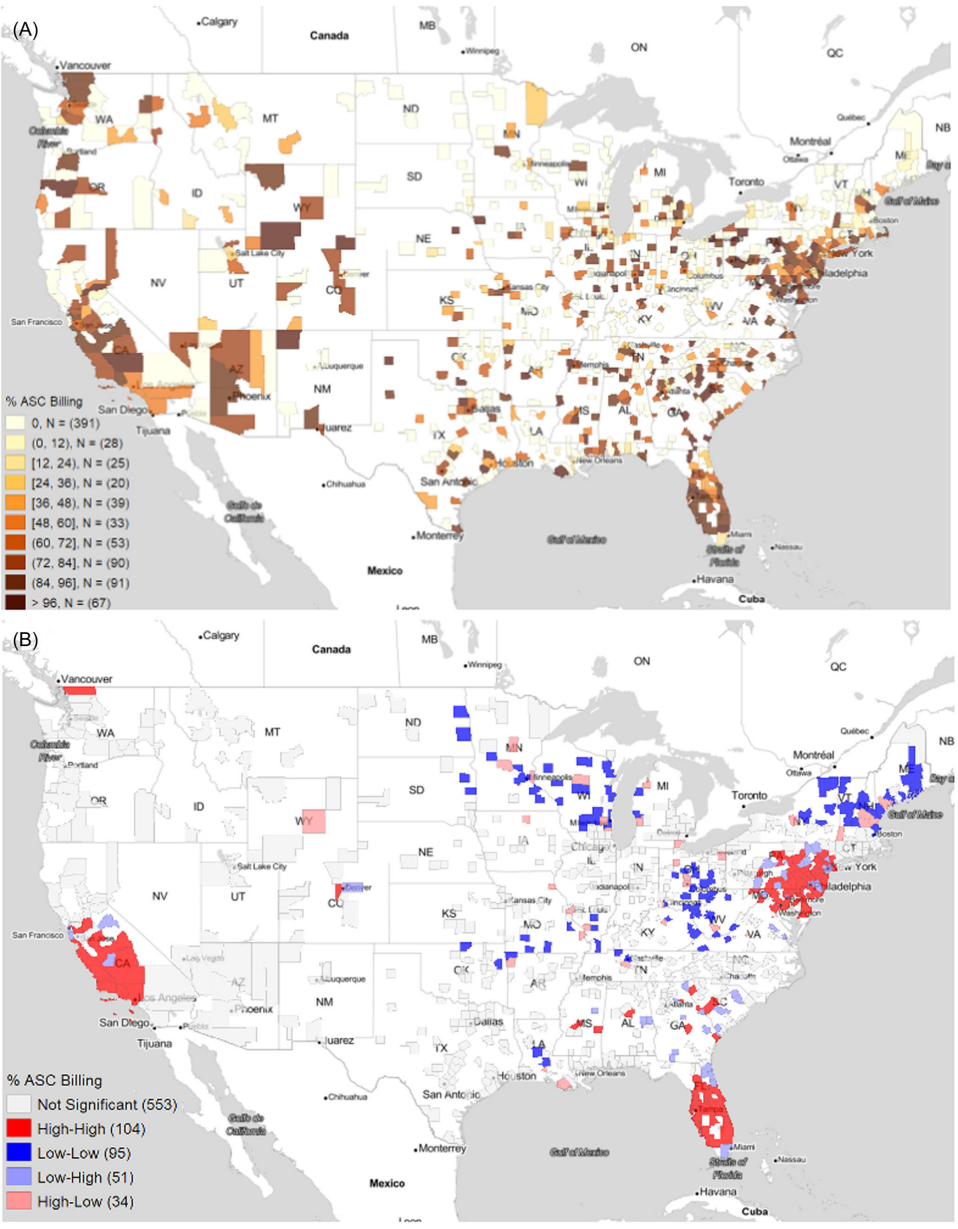
Ambulatory surgery center (ASC) utilization at the county level (A) direct county‐level data of the percent of Medicare funds spent on surgeries performed at ASCs (B) geographic hot spots and cold spots identified using Moran's I statistic. The plotted outcome, “% ASC Billing,” refers to the percentage of total Medicare payment that was from ambulatory surgery centers.

Geospatial clustering analysis of the percent of the top ENT procedures conducted in ASC per county is displayed in Figure 4B with the corresponding percent billing values shown in the adjoining Figure 4A. At *p* ≤ .05, 90 counties were identified as hot spot clusters and 33 as cold spot clusters (Table 3). The counties that were identified as hot spots had an average percent ASC billing of 80.13%. Hot spots were seen in Southern California, Florida, Mid‐Atlantic, and in small, scattered clusters throughout the Deep South. Cold spot clusters had an average percent ASC billing of 2.21% and were located in large swaths of New England/Northeast (Boston, New Hampshire), Ohio, and the Deep South with scattered clusters bisecting the Midwest longitudinally.

**Table 3:** Summary Demographics of All Counties in Each Moran's I Grouping Identified by Geospatial Analysis

Grouping	High‐high (90 counties)			Low‐low (139 counties)			Low‐high (74 counties)			High‐low (33 counties)			Nonsignificant (605 counties)			High‐high to low‐low *t* test
County statistic	10%	Mean	90%	10%	Mean	90%	10%	Mean	90%	10%	Mean	90%	10%	Mean	90%	*p* Value
Number of services: Annual	54.4	1159.1	1185.1	8.7	509.2	1402.8	3.2	1329.6	3206.0	29.9	449.4	933.2	8.2	714.8	1419.2	<.0001
Number of services: Rate^a^	−115.5	66.5	54.8	−193.2	−36.5	96.4	−97.5	5.6	111.2	−99.6	−8.6	44.3	−101.1	−7.1	77.3	.5182
Medicare payment: Annual	16,018	218,758	439,852	799	32,317	82,468	536	110,704	231,875	4393	89,374	182,863	1067	98,026	212,297	<.0001
Medicare payment: Rate^a^	−22,898	11,281	26,467	−11,951	−1522	5963	−6222	659	11,707	−10,511	−1729	13,797	−9349	2862	16,824	.1446
% ASC billing	37.7	69.3	91.0	0.0	1.1	1.8	0.0	2.7	12.2	20.0	54.1	90.5	0.0	22.9	76.2	<.0001
% ASC billing: Rate^a^	−4.9	1.4	8.1	0.0	0.0	0.0	−1.2	−0.5	0.0	−8.7	3.7	19.5	−2.7	0.6	7.0	.0581
% ASC procedures	17.4	51.3	80.3	0.0	0.5	0.4	0.0	0.9	3.3	11.6	39.1	79.0	0.0	16.7	56.6	<.0001
% ASC procedures: Rate^a^	−6.7	1.3	9.3	0.0	−0.1	0.0	−0.1	−0.2	0.1	−7.0	3.0	17.7	−2.8	0.4	5.1	.1160
Average age, y	71.0	72.4	74.0	68.8	70.4	72.0	69.7	71.5	73.0	69.7	71.1	72.2	69.6	71.2	73.0	<.0001
% Male	42.9	44.7	46.8	44.1	46.0	47.5	42.8	45.1	47.4	43.7	45.0	46.9	43.4	45.3	47.4	<.0001
% Non‐Hispanic white	63.5	80.7	92.8	77.0	88.0	96.2	53.5	77.0	94.0	76.9	89.0	95.5	59.7	80.4	95.4	<.0001
% African American	1.3	8.9	19.8	0.3	4.1	10.0	0.7	13.0	31.7	0.5	6.5	17.7	0.4	8.5	24.4	.0019
% Hispanic	0.9	5.8	14.1	0.3	1.2	2.3	0.6	5.7	12.9	0.5	1.4	2.1	0.4	4.1	9.3	<.0001
% Eligible for Medicaid	9.9	16.6	23.6	15.5	23.9	33.7	10.4	19.0	30.0	12.2	19.5	27.4	11.2	19.8	29.1	<.0001
% Poverty	6.9	12.5	19.1	8.4	14.0	20.1	8.3	14.3	23.4	4.9	12.4	18.8	8.1	14.5	21.2	<.0001
Median household income	47,690	64,248	90,205	42,086	54,241	68,665	41,665	59,893	81,988	44,607	61,317	87,789	42,061	56,603	77,498	.0026
Unemployment rate, %	3.6	4.8	6.0	2.9	4.6	6.3	3.2	4.7	6.1	3.1	4.2	5.6	3.1	4.5	5.9	<.0001
% No high school diploma	6.9	11.6	15.1	5.5	10.0	14.5	7.4	12.6	17.9	4.7	8.8	13.0	6.3	11.3	18.0	.0002
% Uninsured	5.9	10.8	16.6	4.6	7.3	11.1	6.7	11.6	16.9	3.9	7.1	10.6	5.7	10.8	16.4	.0095
% Tobacco use	5.0	7.7	10.0	8.2	11.6	16.5	5.7	8.6	11.7	7.5	10.0	13.6	6.2	9.8	13.4	.0008
% Obesity	13.0	19.1	25.1	13.1	18.9	25.7	10.8	17.9	22.8	13.0	18.5	25.4	12.0	17.5	23.8	<.0001
RUCA code	1.00	1.92	3.00	1.00	3.48	6.20	1.00	2.45	4.70	1.00	2.33	4.00	1.00	2.96	6.00	<.0001
Overall population	102,551	547,667	914,269	36,255	139,314	295,239	38,369	364,308	816,765	53,050	266,810	601,164	35,395	270,818	693,456	<.0001
Medicare beneficiaries population	15,824	91,698	143,265	8175	26,068	53,127	7983	54,117	112,863	10,876	46,366	100,964	7176	41,896	95,213	<.0001
Overall population density	129.8	786.6	1906.7	48.1	329.1	564.4	57.4	3579.0	8732.6	88.3	596.6	1666.8	41.7	491.9	1177.2	<.0001
Medicare population density	20.5	134.0	265.7	10.7	59.9	104.5	12.6	484.4	1068.8	16.0	105.2	299.2	8.5	76.8	183.1	<.0001

The rate indicates the annual amount of change as defined by the slope of the fitted regression. Monetary values in USD. 10% and 90% indicate 10th and 90th percentile values among the counties in the group.

Abbreviations: ASC, ambulatory surgery center; RUCA, rural‐urban commuting area (measure of ruralness of a county); USD, $ or US dollar.

^a^ The rate indicates the yearly change from 2015 to 2019 and is calculated as the slope of the fitted regression line.

**Figure 5:**
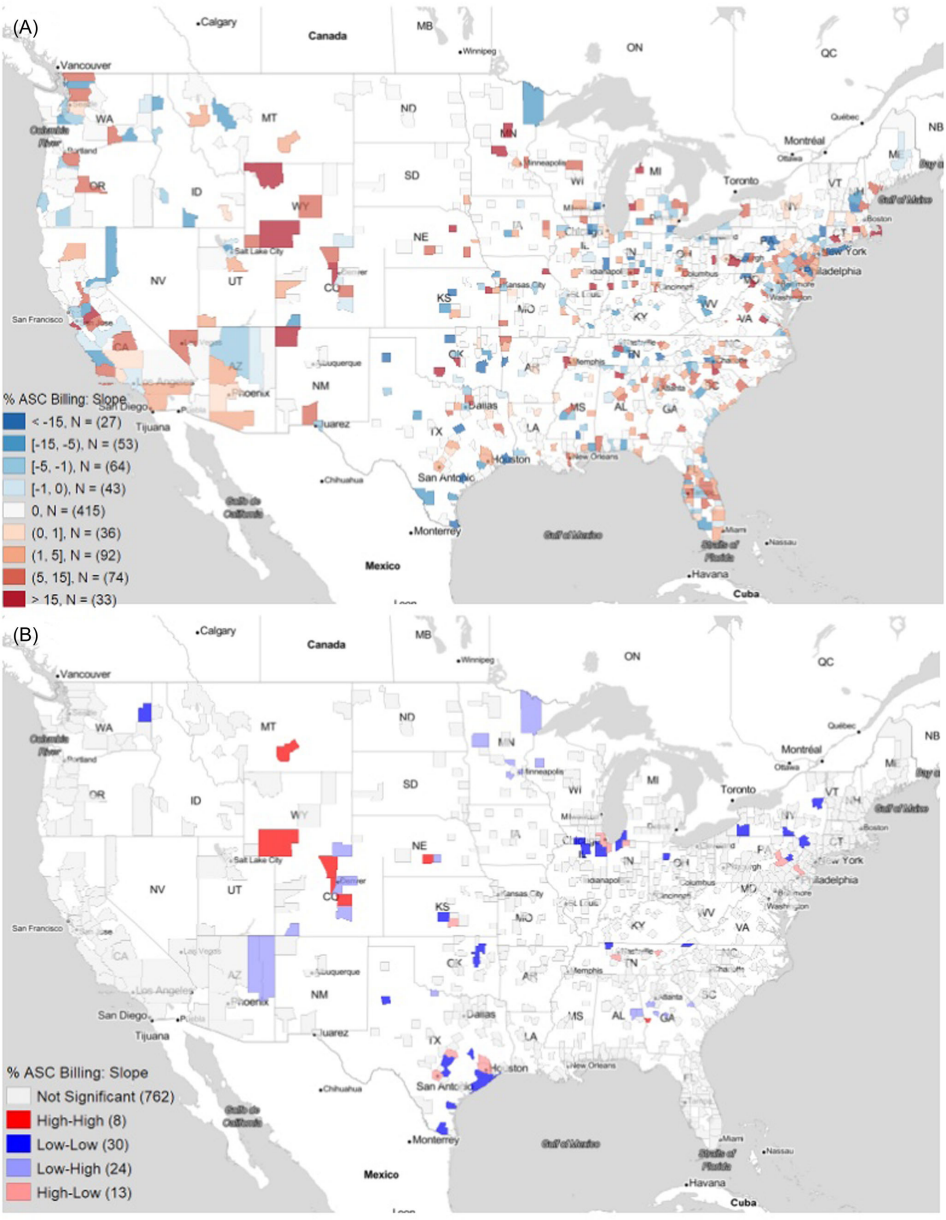
Trends in ambulatory surgery center (ASC) utilization at the county level (A) direct county‐level data of the percent of Medicare funds spent on surgeries performed at ASCs (B) geographic hot spots and cold spots identified using Moran's I statistic. The plotted outcome, “% ASC Billing: Slope,” refers to the yearly increase in percentage of total Medicare payment that was from ambulatory surgery centers. Yearly increase is defined by the slope of the fitted regression lines.

Analysis of the percent change from 2015 to 2019 in ASC billing is displayed in Figure 5. Minimal clustering was seen on a national level. Small hot spots of change in ASC billing were scattered throughout the Rocky Mountains with an average 13.96% increase in billing over the time period. Cold spots, with an average decrease in ASC billing by 1.2%, were dispersed throughout Texas including the Huston metropolitan, the Great Lakes including the Chicago metropolitan, and the Rust Belt.

### Correlates of Geographic Clusters

There were several statistically significant socioeconomic differences between hot spot and cold spot clusters of ASC billing that are displayed in Table 3. Notably, when compared to cold spots, hot spots were older (average Medicare age of 72.4 years old vs 70.4 years old, *p* ≤ .001) and more urban (1.92 vs 3.48 average RUCA classification, a measure of how rural a county is, *p* ≤ .001). Hot spots also had slightly greater rates of unemployment (4.8% vs 4.6%, *p* = .010). Cold spots had a higher percentage of poverty (14.0% vs 12.5%, *p* = .003) and a greater percentage of individuals eligible for Medicaid (23.9% vs 16.6%, *p* ≤ .001). Ethnic and racial factors were also different: hot spots contained greater percentages of African Americans (8.9% vs 4.1%, *p* ≤ .001) and Hispanics (5.8% vs 1.2%, *p* ≤ .001) and lower percentages of non‐Hispanic whites (80.7% % vs 88.0%, *p* ≤ .001).

The main results that are discussed in this section include the geographic denotations of areas of high and low utilization, the geographic locations with the highest and lowest percent change in utilization, and the demographic difference between areas with high and low utilization.

## Discussion

ASCs are a major variable in modern surgical care. Our data shows that utilization of ASCs from 2015 to 2019 was greatest in the western United States, the mid‐Atlantic, and Florida (“hot spots”). Relative to their neighbors, they were least in the Midwest and upper Northeast (“cold spots”). However, the central United States, including both major cities and rural areas, generally had lower utilization than the coasts. Central metropolitan areas like those of Chicago and Houston had a decreasing slope of utilization compared to their neighbors.

The benefit of the utilization of ASC is its cost‐effectiveness and ability to do procedures outside of large hospitals. Though there have been no studies that have investigated the ability of ambulatory surgical centers to improve access to surgical care, we can reasonably expect that the ability to perform outpatient procedures more efficiently could translate to more affordable care and improved operating cost in low‐resource settings. Ideally, this would allow for increased accessibility to care for those populations that have low access to hospital institutions. Medicare reimbursement of ASC has been done as part of the Medicare goal of cost‐cutting and increasing rural involvement. On the contrary, most utilization of ASC currently is in cities in coastal areas that already have high levels of care access and are making the most proportional money compared to their rural counterparts. Among the coastal regions, the upper northeast (Boston) are cold spot regions indicating, that they have significantly lower utilization than their neighbors. Ambulatory surgical centers may allow for increased access to care, especially in cold spots with poor access to hospital care. When looking at safety, it is important to note that these areas consist of vulnerable populations. There is evidence that ambulatory surgical centers can be as safe as or safer than their hospital counterparts. One study looking at Medicare data between 1994 and 1999 found that the overall adverse event rates leading to an emergency department visit were lowest in free‐standing ambulatory‐surgery centers when compared to other counterparts.^9^ It is important to note that there are limitations of patient selection when it comes to ambulatory surgical care. A subsection of patients with comorbidities or poor health will require the setting of a hospital to receive their procedural care.

When examining the demographic data, significant differences were also found between hot spots and cold spots. In the demographic data, starting with age, the hot spots population was a significant 2 years older than the cold spots. There is a trend in the increasing age of the otolaryngology patient population which could impact this difference in age as well.^10^ Individuals in cold spots tended to have lower salaries, and cold spots had a higher percentage of poverty (14% compared to 12.5%) when compared to hot spot areas. These factors impact the access and quality of care patients receive.^11^ When comparing differences in race composition, the cold spots were associated with a greater percentage of non‐Hispanic whites. To note the hot spots tend to be located in more urban areas and the data analysis resolution did not consider intercity variation to access to care. It is possible that different areas of urban locations could have different demographics with different access to care. According to Healthy People 2030, the US government is failing in its goal to reduce the proportion of people unable to obtain needed medical care based on the most recent data published in 2018 to 8.7% from 4.1% in 2017.^12^, ^13^ In the advent of telemedicine, the ability to provide care to patients that would otherwise have to travel long distances to see a primary care physician. As telemedicine develops, access to primary providers will improve. This is especially true as wireless infrastructure continues to develop with government assistance.^14^ As tools to improve access to primary care improves, there is still a need for improvement to simple procedural care. Ambulatory surgical centers could allow for improved access to uncomplicated procedural care. Government subsidies and hospital funding incentives to fund these centers would be an investment in the long term due to their cost‐saving benefits. Also, government‐funded physician incentives to practice in these centers at least part‐time would be beneficial. Note ACS should not replace procedural care for complicated patients that would need more advanced medical infrastructure for their care.

Although we used the top 53 billing codes, the amount of money per code was not evenly distributed and certain billing codes made the vast majority of money (Table 3). The billing codes that were found to make the most were also found to be mostly done in the ASC setting. One interesting point is that the billing code with the most amount of money has a % ASC billing slope that is shallower compared to the other high‐money billing codes. The procedure with the steepest % ASC billing slope is “removal of nasal air passage” (which refers to the submucosal resection of the inferior turbinate), which has been shown to have quite low complication rates in the ASC setting.^15^

There are a few limitations to our study. The first is that the data set is based on Medicare data only, which may not reflect the general population at large. Thus, with the present data, we are cautious to generalize our recommendations to non‐Medicare practice populations. This can be an area for future research. Another limitation is that some counties did not have categorized ENT billing data between 2015 and 2019 associated with them. These counties were sparsely populated. Therefore, we were forced to exclude these counties from the study, and they did not contribute to the determination of hot spots or cold spots. However, geographical areas that do not comprise ENT Medicare billing could have low access to procedures in both ASC and hospital settings. Another limitation is that some areas that have low utilization of ASC could be due to better access to procedures in the hospital setting. In this scenario, these areas would have great access to ENT procedures, but just not in the ASC setting.

## Conclusion

The future of ASC utilization in otolaryngology is multifaceted. As ASC utilization in this field increases, considerations should be made to implement them in areas like those denoted as cold spot locations in this study. This may allow for improved access to this category of care including non‐complex otolaryngological procedures. Although the full extent of the ability of ACS to improve access to surgical care is not fully understood this setting allows for shorter procedures and stays compared to hospital settings assuming that the quality of care is not compromised. These factors could possibly translate to more affordable care in these locations. Future work includes expanding the data sources from Medicare to improve the generalizability of the study. Also, improving the resolution of the study would allow for better investigation of intercity variation to access and their demographic characteristics. Finally, an investigation into the variation of procedures done in the ASC setting in hot spots compared to cold spots should be done to see if there is a significant difference in the types of procedures done.

## Author Contributions

**Mark Nyaeme**, interpreted the geospatial analysis results and drafted the introduction, results and discussion, conclusion, and limitations sections of the manuscript; **Rahul S. Yerrabelli**, interpreted the geospatial analysis results and drafted the introduction, results and discussion, conclusion, and limitations sections of the manuscript, performed the created code, Moran's statistical analysis, produced all figures, and produced all tables; **Nicholas Peterman**, extracted the data from publicly available sources, compiled it into a machine‐readable format and drafted the methods; **Brad Kaptur**, edited the manuscript, and contributed to the conception and planning of the project; **Eunhae Yeo**, edited manuscript and contributed to the conception and planning of the project; **Kristine R. Carpenter**, is a practicing physician and provided clinical insight, edits to the manuscript, and supervision of the project.

## Disclosures

### Competing interests

The authors deny any financial or ethical conflicts of interest. Specifically, no authors have financial ties or ownership of surgical centers.

### Funding source

The authors declare that no funds, grants, or other support were received specifically for the preparation of this manuscript.

## Supporting information

Supplemental File 1. All 53 analyzed ENT procedures by Medicare payment with linear regression analysis of key metrics from 2015 to 2019. This is an extended version of Table 1.

## Data Availability

The original data sets used are available publicly. The compiled, machine‐readable formatting of the data set is available from the corresponding author on request and will be made publicly available after the publication of this manuscript.
